# Electron Beam Irradiation Cross-Linked Hydrogel Patches Loaded with Red Onion Peel Extract for Transdermal Drug Delivery: Formulation, Characterization, Cytocompatibility, and Skin Permeation

**DOI:** 10.3390/gels9010052

**Published:** 2023-01-07

**Authors:** Pimpon Uttayarat, Rattanakorn Chiangnoon, Thanu Thongnopkoon, Kesinee Noiruksa, Jirachaya Trakanrungsie, Wattanaporn Phattanaphakdee, Chuda Chittasupho, Sirivan Athikomkulchai

**Affiliations:** 1 Nuclear Technology Research and Development Center, Thailand Institute of Nuclear Technology (Public Organization), Nakhon Nayok 26120, Thailand; 2 Department of Pharmaceutical Technology, Faculty of Pharmacy, Srinakharinwirot University, Nakhon Nayok 26120, Thailand; 3 Department of Pharmacognosy, Faculty of Pharmacy, Srinakharinwirot University, Nakhon Nayok 26120, Thailand; 4 Department of Pharmaceutical Chemistry, Faculty of Pharmacy, Srinakharinwirot University, Nakhon Nayok 26120, Thailand; 5 Department of Pharmaceutical Sciences, Faculty of Pharmacy, Chiang Mai University, Chiang Mai 50200, Thailand

**Keywords:** *Allium cepa*, wound healing, transdermal delivery, hydrogel, electron beam irradiation

## Abstract

The use of bioactive molecules derived from medicinal plants in wound healing has recently attracted considerable attention in both research and public interest. In this work, we demonstrated the first attempt to incorporate the extract from Thai red onion skins in hydrogel patches intended for transdermal delivery. The red onion skin extract (ROSE) was first prepared and evaluated for cytotoxicity by MTT assay with both L929 and human dermal fibroblast cells. Hydrogel patches with porous microstructure and high water content were fabricated from polyvinyl alcohol (PVA) by electron beam irradiation and characterized for their physical, mechanical, morphological, and cytocompatible properties prior to the loading of ROSE. After decontamination by electron beam irradiation, the in vitro release profile exhibited the burst release of extract from ROSE-coated hydrogel patches within 5 h, followed by the sustained release up to 48 h. Finally, evaluation of skin permeation using Franz cell setup with a newborn pig skin model showed that the permeation of ROSE from the hydrogel patch increased with time and reached the maximum of 262 µg/cm^2^, which was well below the cytotoxicity threshold, at 24 h. These results demonstrated that our ROSE-coated hydrogel patches could potentially be used in transdermal delivery.

## 1. Introduction

Injury to the skin can happen throughout the lifetime of a person, which may span from a minor cut that takes a relatively short time to heal to a substantial wound that takes a much longer time to close. The natural wound healing process typically occurs immediately following the lesion until complete skin restoration. This complex but highly orchestrated process can be characterized by a series of overlapping phases: (1) hemostasis, (2) inflammation, (3) proliferation, and (4) remodeling [1]. When these phases are interrupted and the wounds are left open for more than a month, chronic wounds may develop, posing health risks to patients [2,3,4]. However, for wounds that heal with excessive overgrowth of skin and connective tissue, unaesthetic hypertrophic scars may occur [5]. Therefore, it is desirable to have natural wound healing progress through every phase without delay or to not have it result in any scarring by the end.

Plants with bioactive molecules have long been recognized for their medicinal benefits in skin wound healing and have recently become the focus of research in chronic wound healing as well as the management of keloids and hypertrophic scars [6,7,8,9]. Among these, *Allium cepa* L., which belongs to the Liliaceae (Amaryllidaceae) family and is commonly known as onion, used in culinary preparations worldwide, has emerged as a candidate to treat skin wounds as well as keloids and hypertrophic scars [10,11,12,13,14]. Enriched with various bioactive molecules such as vitamins, minerals, flavonoids, organosulfur, and phenolic compounds [15], onion provides various therapeutic effects including antimicrobial, antioxidant, anticancer, anti-inflammatory, and antidiabetic effects that are beneficial for the digestive, circulatory, respiratory, and immune systems [15,16].

For wound healing purposes, various topical gels containing onion extract are available as over-the-counter products for scar healing, such as Mederma^®^ and Contractubex^®^ [17]. Previous clinical evaluations demonstrated that Contractubex^®^ reduced the increase of scar width and lowered the occurrence of hypertrophic scars and keloids [17]. Likewise, applying onion extract in topical gels could relieve itching and pain and improve the softness, redness, and texture associated with keloids and hypertrophic scars [11,13,18]. In addition, treating burn wounds in rat models with poultices containing *A. cepa* could improve wound healing activity by reducing wound size and damage produced by burns [14].

Transdermal drug delivery patches based on hydrogels are widely used for the transdermal delivery of various drugs [19]. Transdermal delivery allows patients to self-administer medications in a painless and convenient manner. The hydrogel patch is one of the transdermal delivery systems that is a desirable form of drug delivery because of the obvious advantages over topical gel preparation. Hydrogels can treat both exudating and dry necrotic wounds [1,20,21]. Hydrogels also have other distinguishing characteristics such as softness, malleability, and biocompatibility. Because of their porous and hydrated molecular structure, hydrogels are one of the candidates with the greatest potential to mimic the native skin microenvironment. They can be used as a permanent or temporary dressing for various wounds to help the injured epidermis, dermis, or both regenerate and heal [22]. Compared with the topical conventional gel, the hydrogel patch eliminates the need for frequent dosing and the overdosing drug plasma level, while maintaining a constant drug concentration [23]. The hydrogel patch was easier to use, and there was less contamination from the direct touch of the product. The dose of the drug was consistent in each patch. Most of the hydrogel patches were non-adhesive and easy for removal. All of these contribute to improved patient compliance, particularly when long-term treatment is required. Transdermal systems are generally less expensive than other therapies because patches are designed to deliver drugs for 1 to 7 days.

The beneficiary benefits of onion in wound healing and scar management are believed to stem from quercetin, the flavonoid component predominant in the onion peel [24,25,26]. Recent studies have shown that quercetin promoted diabetic wound healing by switching macrophages from type M1 to M2 [27], reduced the nitric oxide release as well as pro-inflammatory cytokines in macrophages [16], mediated the reduction of fibrosis associated with scar formation [5], and inhibited the excessive fibroblast proliferation and collagen production that results in scarring [24,28]. The onion peel itself is non-edible and disregarded from consumption [16]; therefore, the use of onion peel as a starting material to formulate bioactive wound dressings is considered value-added and beneficial to wound healing and scar management.

Red onion skin extract (ROSE) contains a variety of bioactive compounds including sulfur-containing compounds such as onionin A, cysteine sulfoxides, and flavonoids, mainly quercetin and its glucosides [29]. Quercetin is the major compound in the skin of red onion [15,25,30]. Quercetin is reported to be effective in treating a variety of diseases, including wounds, scars, and keloids [31]. The effective doses of quercetin for wound healing were reported in [32,33,34]. Quercetin at concentrations of 1.5–6.0 mg/mL has been shown to promote proliferation and migration of fibroblasts and enhance cutaneous wound healing capacity in mice [31]. Ahmad reported that the quercetin ointment (1%) resulted in a significant increase in wound contraction by enhanced epithelization, and the possible mechanism of improved healing by quercetin is due to the ability of quercetin to improve the tissue’s antioxidant levels [33]. Kant et al. have shown that 0.1% and 0.3% quercetin accelerate wound healing efficiently by modulating the antioxidant system of wounds, cytokines, growth factors, and other proteins and cells involved in healing [34].

Hydrogels are soft materials consisting of three-dimensional (3D) networks of hydrophilic polymers and water that fills the network space [35]. Among various types of wound dressings, hydrogels have gained considerable attention as a new class of dressing materials due to their ability to provide appropriate moisture for wound healing and softness similar to the skin [4]. The 3D networks of hydrogels can be crosslinked by physical, chemical, or irradiation methods [36]. While the physical method can be easily achieved by film casting or freeze–thawing techniques, which results in a reversible gel structure [36], the permanent gel structure can be obtained by chemical and irradiation methods. Using ionizing radiation from high-energy sources such as gamma rays or electron beams to crosslink hydrogels is considered advantageous, as no additional chemical crosslinkers or initiators are required, which can be harmful to the body if their excess is left inside the hydrogels [37]. In addition, sterilization of the final products can be combined with the processing step [37]. Various synthetic and natural polymers such as polyvinyl alcohol (PVA) [38,39], polyvinyl pyrrolidone (PVP) [40,41], polyethylene oxide (PEO) [40,42], chitosan [41], bacterial cellulose [43], gum [44], and pectin [45] have been used to form crosslinked hydrogels by electron beam irradiation.

The use of hydrogel dressings incorporated with onion extract for dermal applications has been previously reported. Pagano et al. showed the sustained release profile of red onion skin extract from hydrogel films prepared from PVP and carboxy methyl cellulose by film casting up to the maximum concentration of 0.69 mg/mL at 48 h [16]. This concentration was demonstrated to be non-cytotoxic to HaCaT cells while providing anti-bacterial and anti-inflammatory activities necessary in wound treatment. In managing hypertrophic scars and keloids, Campanati et al. showed that the medical-device grade, an adhesive overnight patch containing onion extract and other active ingredients, could improve the clinical and morphological characteristics of a scar when applied for 8 h once a day [46]. With these growing interests in the use of onion extract in dressings for wound treatment and management, different methods that can fabricate and decontaminate ready-to-use dressings containing onion extract will be of technological importance.

Our previous work has identified the presence of quercetin in the extract derived from Thai red onion skins by reverse phase HPLC technique [26], which could support the potential use of Thai red onion skins in wound healing and scar management. It was shown that a ROSE-coated hydrogel patch contains 567.73–784.33 µg quercetin per gram of hydrogel patch. The ROSE-coated hydrogel patch could be applied for wound healing as it contains quercetin in a therapeutic dose. In this present study, we showed for the first time the development of decontaminated hydrogel patches incorporated with Thai red onion skin extract (ROSE) by electron beam irradiation and the evaluation of the release profile, as well as transdermal permeation of the extract from hydrogels for their applications in transdermal delivery. PVA was chosen to fabricate hydrogels due to its biocompatibility, non-toxicity, and water solubility [47]. The hydrogel patches were first formed by electron beam irradiation of starting PVA solution and characterized for their swelling, rehydration, porous microstructure, tensile properties, and cytocompatibility. Extract derived from the skins of Thai red onion was then evaluated for its non-cytotoxic concentration by MTT assay with both L929 and HDFB cells before being coated on hydrogel patches. After decontamination by electron beam irradiation, the release of extract from ROSE-coated hydrogel patches was determined in vitro by a UV–visible spectrophotometer, and transdermal permeation was evaluated using a pig skin model.

## 2. Results and Discussion

### 2.1. The Yield of Thai ROSE

The yield of extract obtained from dried *A. cepa* skins was measured gravimetrically. The yield was reported as 10.58% *w*/*w*. The yield of ROSE obtained from this study was comparable to that reported by Pagano et al. (9.7% *w*/*w*) [16].

### 2.2. Physical Properties of Hydrogels

**Figure 1:**
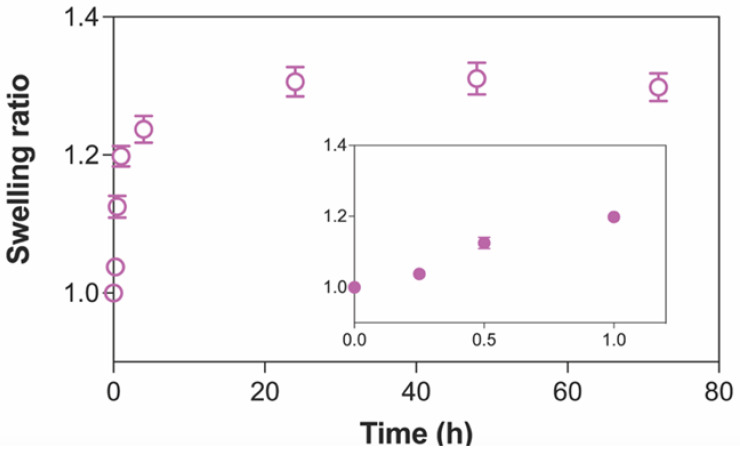
The kinetics of swelling of PVA hydrogels crosslinked by electron beam irradiation over 72 h. Inset shows the rapid swelling behavior at initial time points. Data are presented as mean ± standard deviation (*n* = 4).

Swelling and gel fraction are the two fundamental aspects of hydrogels that relate to their abilities to absorb fluid while retaining their integrated 3D structure. Because our hydrogel patches composed of 10% (*w*/*v*) PVA (10PVA) will be applied in their hydrated state, the swelling of additional fluid into the hydrogel matrix was characterized by the swelling ratio in which the swollen weight was normalized by the initial weight of hydrogel patch in the hydrated state. Figure 1 shows that the swelling ratio of 10PVA hydrogel patches increased rapidly from 1 to 1.2 within one hour after immersion in an aqueous environment. After 4 h of immersion, the swelling ratio increased slightly before being ceded at 24 h. At equilibrium, the maximum swelling ratio of our 10PVA hydrogel patch remained at 1.3, which was relatively within the same range as the previous study [48]. These data suggested our hydrogel patches were capable of imbibing 30% more fluid into the already hydrated structure, and the maximum swelling capacity was obtained within 24 h.

Table 1 summarizes the total capacity of hydrogel patches to imbibe water compared to their dried state, the portion of stable gel structure, and the moisture retention property represented by EDS, gel fraction, and *R_h_*, respectively. The high percentage of the water content, about 93%, in our hydrogel system was similar to the value reported in a previous study for PVA hydrogels formed by gamma irradiation [48]. Compared to its dried weight, the EDS of our 10PVA hydrogel patches was ~1700%. Regarding gel fraction, the stable gel portion of our crosslinked 10PVA hydrogel patches of about 90% was within the same range as previously reported data for PVA hydrogels crosslinked by gamma or electron beam irradiation [35,49,50]. This high percentage of gel fraction in our PVA hydrogel patches compared to those formed by physical methods such as casting suggested that ionizing radiation could effectively induce most of the polymer chains in the starting aqueous solution to be chemically crosslinked into 3D networks [51]. For the ability of hydrogels to retain moisture within the structure, our data showed that the moisture retention capability, or *R_h_*, decreased sharply by half after 2 h before it reached ~10% at 24 h. This reduction in *R_h_* of our PVA hydrogel patches to 10–20% within 24 h was similar to other hydrogel systems, such as PVP [52] and PVA/chitosan/gelatin [53], which were also crosslinked by irradiation. However, the sharp reduction in *R_h_* in our systems within the first two hours can be attributed to the differences in hydrogel compositions and relative humidity used in the experiments compared to previous reports [52,53]. Nonetheless, for the practical use of hydrogels as sheet dressings for an extended period of time without daily changes, it may require a cover film over hydrogels to prevent water loss [54].

### 2.3. Mechanical Properties

**Figure 2:**
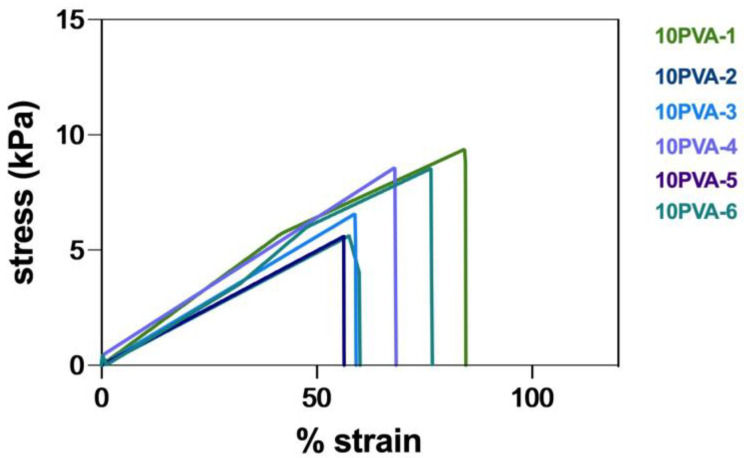
The stress–strain curves of 10PVA hydrogel patches (*n* = 6).

Based on the tensile analysis, the stress–strain curves of all 10PVA hydrogel patches exhibited a linear trend with similar slopes as the samples were extended under loading until breaking (Figure 2). The single slope along the stress–strain curve corresponded to the chemically crosslinked 3D networks formed by irradiation, which served as the only structural support for the hydrogel matrix. Across all five samples, the tensile strength varied in the range of 5–10 kPa, and the elongation at break was within 50–90% strain. As summarized in Table 2, the average tensile strength, elongation at break, and Young’s modulus shown in this present study were similar to those obtained from PVA hydrogels processed by gamma irradiation at the same dose [35,55]. In addition, these values were also within the soft tissue regime of skin proposed in the literature [56].

### 2.4. Cytocompatibility of Hydrogel Patch 

**Figure 3:**
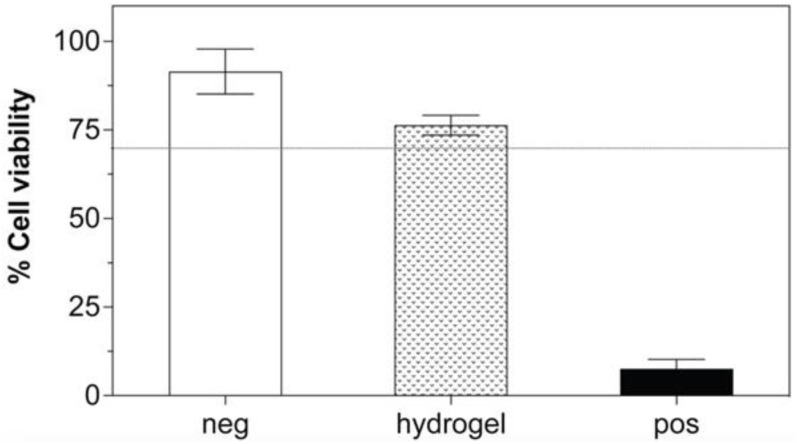
Cytotoxicity evaluation of hydrogel sheets on L929 by MTT assays (*n* = 3). The dashed line represents 70% cell viability. Negative (neg) control refers to high-density polyethylene, and positive (pos) control refers to polyurethane containing 0.1% zinc.

Using the MTT-based extract test, the cell viability after treatment with extract eluted from non-treated 10PVA hydrogel patches was 75%, whereas it was 90% and 7%, respectively, for the negative and positive controls (Figure 3). As the cell viability of our hydrogels was above the 70% threshold for the tested materials to be considered non-cytotoxic [57,58], our results indicated that the 10PVA hydrogel patches fabricated by electron beam irradiation were cytocompatible. Our cytocompatibility results also agreed with previous reports that evaluated PVA-based hydrogels crosslinked by electron beam [59] and gamma irradiation [57], suggesting that ionizing irradiation from high-energy sources provided a safe crosslinking method of hydrogels for biomedical use. In addition, the elution process of hydrogels for the 24 h required for skin-contact surface devices confirmed the potential use of our hydrogel sheets as skin wound dressings [49].

### 2.5. Morphology of ROSE-Coated Hydrogel Patch

**Figure 4:**
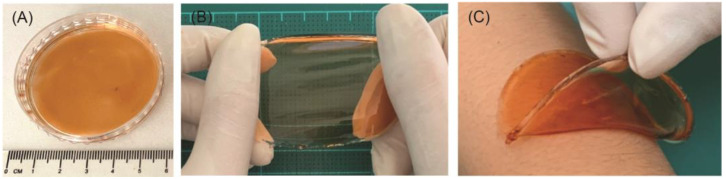
Digital images of (**A**) ROSE-coated hydrogel patch showing its (**B**) stretchability and (**C**) ability to conform to skin.

The extract derived from Thai red onion skins was uniformly coated on a 10PVA hydrogel patch (Figure 4A). These hydrogel patches containing the red onion extract, or ROSE-coated hydrogel patch, were shown to be stretchable and conformal over skin contour (Figure 4B,C).

**Figure 5:**
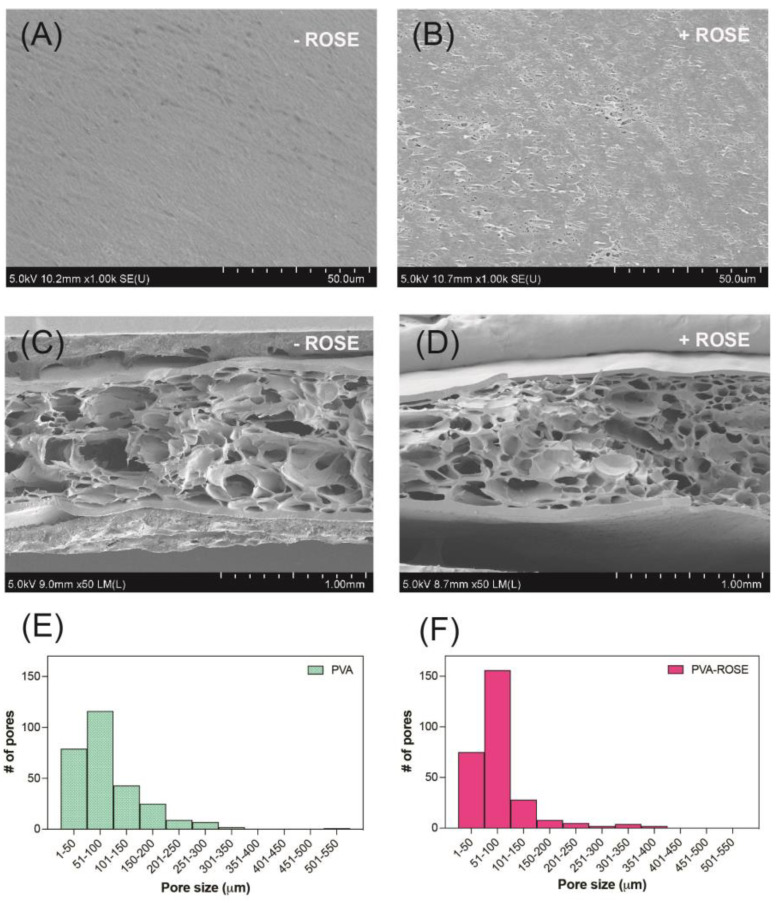
SEM images of 10PVA and ROSE-coated hydrogel patches. (**A**,**B**) Top views of hydrogel surfaces. (**C**,**D**) Cross sections of hydrogels. (**E**,**F**) Histograms showing the distribution of pore size (*n* = 250–300) in (**C**,**D**), respectively. The symbols ‘−’ and ‘+’ indicate that the hydrogels were without and with ROSE coating.

As electron beam irradiation induced PVA molecules to crosslink into 3D networks, the resulting porous structure inside the hydrogel matrix was analyzed by SEM (Figure 5). From the top view (Figure 5A,B), both 10PVA and ROSE-coated hydrogel patches showed relatively flat surfaces with some small pores sparsely formed on the ROSE-coated sample. By contrast, the heterogeneous distribution of micro-scale pores was observed throughout the entire matrices of both hydrogels (Figure 5C,D), confirming the presence of crosslinked 3D networks. Analysis of pore size (histograms, Figure 5C,D) showed that both hydrogels had a similar distribution of pores in which the average diameter of most pores was within the range of 1–50 and 51–100 µm, with a few larger ones up to 550 µm. Therefore, the incorporation of extract did not alter the porous structure of ROSE-coated hydrogels.

### 2.6. Effect of ROSE Coating on Swelling and Mechanical Properties of Hydrogels

**Figure 6:**
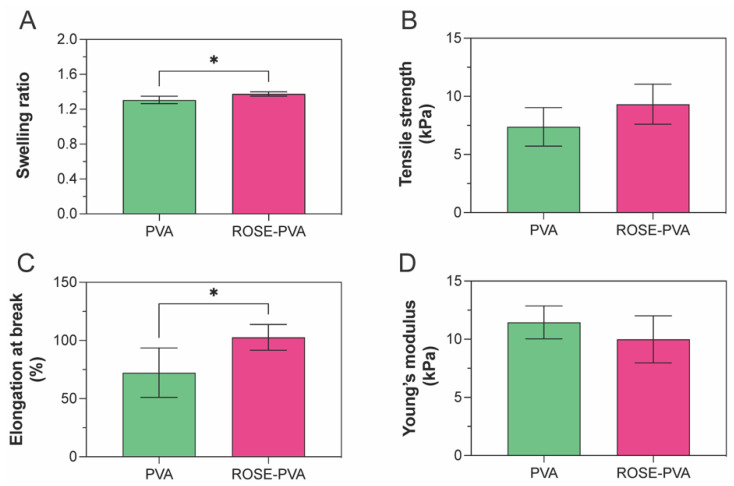
Effect of ROSE coating on (**A**) swelling, (**B**) tensile strength, (**C**) elongation at break and (**D**) Young’s modulus of hydrogel patches. The swelling data are collected from 4 replicates and the mechanical properties data are collected from 6 replicates. The symbol ‘*’ indicates *p*-value < 0.05.

At 24 h, where the swelling reached equilibrium, the ROSE-coated hydrogel patches showed ~5 % increase in swelling ratio compared to 10PVA hydrogels (Figure 6A). This can be due to the presence of bioactive molecules such as phenolic compounds in ROSE that attract water molecules through hydrophilic side chains into the hydrogel matrix. Regarding mechanical properties, both the tensile strength and Young’s modulus of ROSE-coated hydrogels remained similar to 10PVA, while the elongation at break increased ~40% (Figure 6B–D). The increase in elongation at break could be attributed to secondary forces such as hydrogen bonding between hydrophilic groups on bioactive compounds in ROSE and the –OH side chains of the PVA matrix that might help with bridging adjacent PVA chains together. However, these secondary forces are not enough to significantly raise the tensile strength and Young’s modulus of ROSE-coated hydrogel patches.

### 2.7. Decontamination of ROSE-Coated Hydrogel Patch

Two doses of electron irradiation, 5 and 10 kGy, were tested to decontaminate ROSE-coated hydrogel patches. The plate count method confirmed the absence of any detectable bacterial colony after the second irradiation compared to the non-irradiated ones. Based on these data, the 5-kGy dose was chosen for the decontamination of ROSE-coated hydrogel patches (Table 3).

### 2.8. In Vitro Release Profile of ROSE from Hydrogel Patch

**Figure 7:**
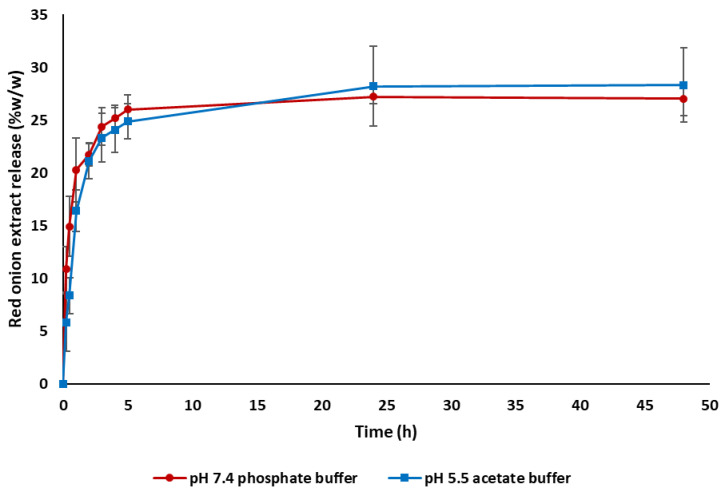
Cumulative release profiles of ROSE from hydrogel patch in phosphate-buffered saline pH 7.4 and acetate buffer pH 5.5 (*n* = 3).

The release study of the red onion extract from the ROSE-coated hydrogel patches was carried out in phosphate buffer saline (pH 7.4) and acetate buffer (pH 5.5). The release profiles of ROSE from the hydrogel are shown in Figure 7. The results showed that the release profiles of red onion extract were comparable at pH 7.4 and pH 5.5, suggesting that pH did not affect the release behavior of the extract from the hydrogel patch. The red onion extract was rapidly released in the first hour, and the release rate was then gradually slower before reaching the plateau. Within 5 h, the ROSE revealed fast release with the amount of 1.02 ± 0.06 mg (26.02 ± 1.40% *w*/*w*) and 0.97 ± 0.07 mg (24.89 ± 1.69% *w*/*w*) at pH 7.4 and pH 5.5, respectively.

This release behavior conformed to the previous studies involving chemically crosslinked PVA hydrogel [60] and Carbopol-based hydrogel [61,62]. The burst release in the initial stage might be due to the surface-bound drug, including the free drug dissolved and diffused through channels of the porous structure of the PVA hydrogel patch. Subsequently, the swollen PVA hydrogel might gradually narrow the channels, leading to the retarded drug release profile. This is related to the swelling kinetics of PVA hydrogel in Figure 1. The initial burst release and subsequent sustained release might be beneficial for the antimicrobial and anti-inflammatory activities of ROSE to manage wound healing. The burst release of ROSE resulted from the coating of ROSE on the surface of the hydrogel patch, which might be minimized by entrapment of ROSE in the hydrogel matrix or encapsulation in the micro/nanoparticles [63].

### 2.9. Permeation of ROSE from Hydrogel Patch through Newborn Pig Skin

**Figure 8:**
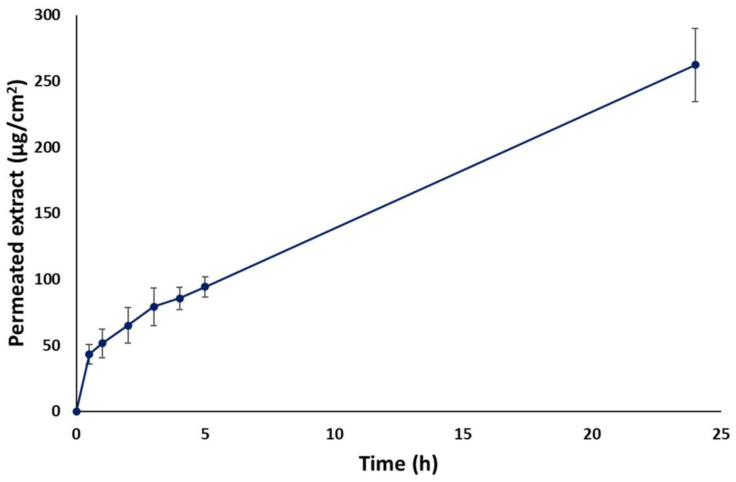
In vitro skin permeation of ROSE from hydrogel patch through newborn pig skin (*n* = 3).

The cumulative amount of ROSE permeated through the newborn pig skin is presented in Figure 8. The amount of ROSE permeated increased with increasing incubation time. The greatest permeation was observed where 262.28 ± 27.63 µg/cm^2^ ROSE permeated from the donor chamber through the newborn pig skin into the receptor medium within 24 h. The continuous permeation of the extract through newborn pig skin might involve skin hydration by water, which behaves as a permeation enhancer [64,65]. Likewise, PVA has been reported as a permeation enhancer by interaction with lipids and keratin in the stratum corneum [65]. In addition, quercetin, a major bioactive compound in red onion peel itself, showed significant permeability through the human skin [66].

### 2.10. FTIR Spectra of ROSE-Coated Hydrogel Patch

**Figure 9:**
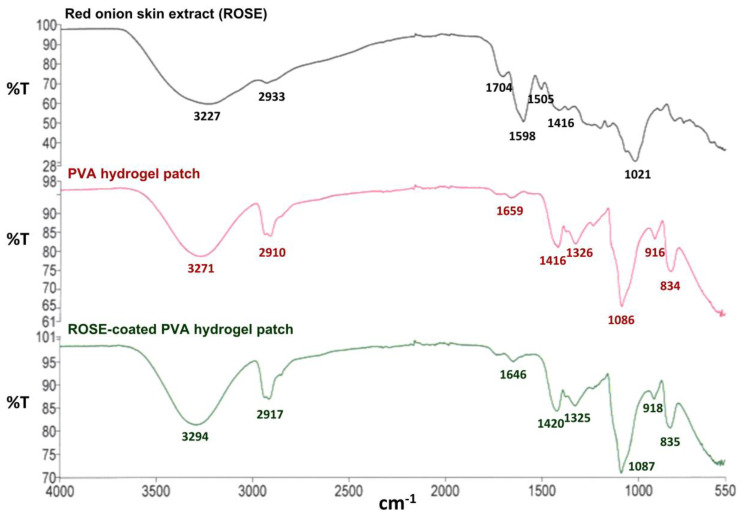
FTIR spectra of ROSE, PVA hydrogel patch, and ROSE-coated PVA hydrogel patch.

FTIR spectra of red onion skin extract (ROSE), PVA hydrogel patch, and ROSE-coated PVA hydrogel patch are shown in Figure 9. The FTIR spectrum of ROSE revealed two bands of OH stretching of hydroxyl group and CH stretching of methylene at 3227 and 2933 cm^−1^, respectively [67]. Three distinctive peaks at 1704, 1598, and 1021 cm^−1^ were attributed to the presence of carbonyl (C=O) group, double bond (C=C) aromatic ring stretching, and C–C stretching or ether group, respectively. The FTIR spectrum of ROSE in this study was comparable to that examined by Verma et al. and Ibezim-Ezeani MU et al. [67,68]. The characteristic peaks also relatively related to the structure of quercetin, which is a main ingredient in skin onion extract. The FTIR spectrum of PVA hydrogel revealed two important peaks at 3271 and 2910 cm^−1^ which related to the OH stretching of the hydroxyl group and CH stretching of the alkyl group, respectively [60]. Regarding the FTIR spectrum of the ROSE-coated PVA hydrogel patch, the major shift of the peak corresponding to OH stretching of the hydroxyl group between 3200 and 3300 cm^−1^ could be observed, indicating the potentially formed molecular interaction, e.g., hydrogen bonding, between hydroxyl groups of PVA and certain compounds of ROSE. In addition, the molecular interaction may impact the physical properties of hydrogel patches and cause the detainment of ROSE after the burst release in the in vitro release study.

### 2.11. Cytocompatibility of ROSE at a Varied Concentration

**Figure 10:**
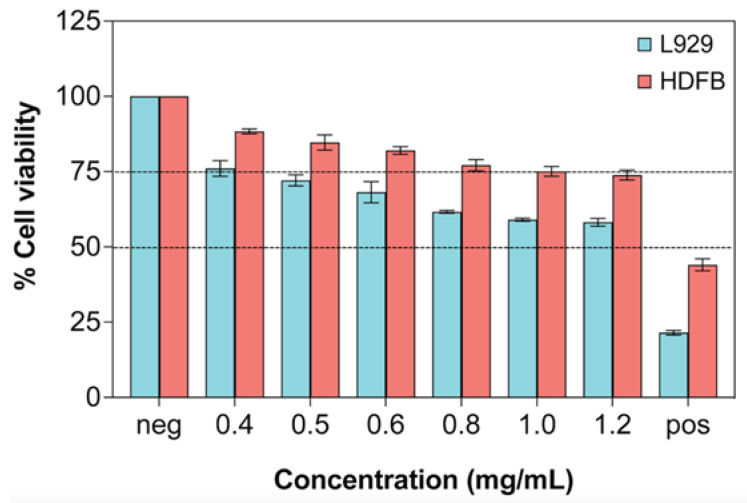
Cytotoxicity evaluation on L929 and HDFB cells after treatment with ROSE for 24 h by MTT assay (*n* = 3). Dashed lines represent 50% and 75% cell viability. Negative (neg) control is complete media, and positive (pos) control is complete media with 10% (*v*/*v*) DMSO.

Based on the MTT assay, the cell viability of both L929 and HDFB cells was above 50% at all ROSE concentrations from 0.4 up to 1.2 mg/mL. Between the two cell lines, L929 seemed to be more sensitive to the extract concentration as the cell viability sharply decreased to 75% at the lowest concentration and further decreased to 58% at the highest concentration (Figure 10). By contrast, the viability of HDFB slightly decreased with the extract concentration and remained ~75% at the highest extract concentration. Compared to the previous report by Pagano et al. that evaluated the cytotoxicity of onion extract in HaCaT and RAW 264.7 cell lines by MTT assay [16], the viability of both cell lines remained above the 75% threshold at the extract concentration range of 0.015–0.5 mg/mL. Therefore, using the same 75% cell viability threshold, the safe concentration of ROSE in our study was 0.4 mg/mL, similar to the previous report [16]. In addition, the transdermal permeation study in our study showed that about 262 µg of the extract could permeate through 1 cm^2^ of pig skin within 24 h. Therefore, this amount of permeated extract was well within the safe concentration.

## 3. Conclusions

In this report, we demonstrated the fabrication and characterization of hydrogel patches coated with Thai red onion skin extract for transdermal delivery. The extract from Thai red onion skins was obtained with a high yield. Electron beam irradiation was applied to form hydrated, cytocompatible hydrogel patches with a porous microstructure that was stretchable and conformal to the skin. After loading with red onion skin extract and radiation decontamination, the hydrogel patches were shown to release the extract, which could permeate through the pig skin model at a concentration well below the cytotoxicity level.

## 4. Materials and Methods

### 4.1. Materials

PVA (Mw 89,000–98,000), 3-(4,5-Dimethylthiazol-2-yl)-2,5-diphenyl tetrazolium bromide (MTT), isopropanol, fetal bovine serum (FBS), Eagle’s minimal essential medium (MEM), Dulbecco’s Modified Eagle’s medium (DMEM), tissue-culture grade water, L-glutamine, penicillin streptomycin, and dimethyl sulfoxide (DMSO) were purchased from Sigma-Aldrich (Saint Louis, MO, USA). L929 mouse fibroblasts (NCTC clone 929) and human dermal fibroblasts (HDFB, PCS-201-012) were purchased from ATCC (Manassas, VA, USA). Polyurethane containing 0.1% zinc (positive control) and high-density polyethylene (negative control) was purchased from Hatano (Kanagawa, Japan). Deionized (DI) water was used to prepare solutions in all experiments.

### 4.2. Plant Material and Extraction

Red onions (*Allium cepa*) were purchased from Chiangmai, Thailand, in 2021. Plant material was identified by Dr. Sirivan Athikomkulchai, and the specimen (SIRA004) was kept at the Faculty of Pharmacy, Srinakharinwirot University. The extraction method was adapted from Pagano et al. [16]. After washing and naturally drying, the outside layer of red onions was peeled and ground into fine powders for extraction. The plant extraction was performed by using 70% ethanol at a ratio of 1 g of powders and 40 mL of 70% EtOH at 60 °C for 90 min. The extract was filtered and dried by using a rotary evaporator. After evaporation, the extract was suspended in ultrapure water (1 g of extract and 7 mL of water). The water extract was centrifuged at 20 °C for 20 min at 4000 rpm. The obtained supernatant was freeze-dried and kept at −20 °C before use. The percentage of red onion skin extract (ROSE) yield was calculated by the following equation:

*Yield (%) = (Amount of freeze dried extract)/(Amount of dried plant) × 100%* (1)

### 4.3. Preparation of PVA Hydrogel Sheets by Electron Beam Irradiation

A 10% (*w*/*v*) PVA solution was first prepared by dissolving PVA powder in hot boiling DI water and homogeneously mixed by a magnetic stirrer for 30 min at 80 °C. After the solution was cooled to room temperature, it was transferred to 5.5 cm diameter Petri dishes with closed lids. Irradiation of samples was carried out at the Irradiation Center, Thailand Institute of Nuclear Technology (Public Organization), using an MB10-50 linear electron accelerator with an energy of 10 MW to obtain a total dose of 40 kGy. The resulting PVA hydrogel sheets were refrigerated before coating with ROSE.

### 4.4. Characterization of Swelling, Gel Fraction, and Water Retention

After being crosslinked by electron beam irradiation, the entire hydrogel samples were immersed directly in solution A, which simulated the exudate at wounds as adapted from European Standard EN 13726 [35]. At designated time points from 15 min up to 72 h, the samples were blotted and weighed. The swelling ratio of hydrogels was calculated by the following equation:

*Swelling ratio = W_t_/W_i_* (2)

where *W_t_* is the swollen weight at a specified time point, and *W_i_* is the initial weight of hydrogels. Experiments were performed in 4 replicates.

At the termination of the swelling experiment, all samples were dried at 60 °C to a constant weight (*W_d_*). Then, the equilibrium degree of swelling and water content was calculated by

*EDS (%) = (W_s_ − W_d_)/W_d_ × 100%* (3)

*Water content (%) = (W_i_ − W_d_)/W_i_ × 100%* (4)

where *W_s_* is the swollen weight at equilibrium.

To determine the stable gel portion of the crosslinked 3D network, the samples were boiled for 15 min before being dried to a constant weight (*W_f_*) at 60 °C. Then, the gel fraction was calculated by the following equation:

*Gel fraction (%) = W_f_/W_d_ × 100%* (5)

Experiments were performed in 4 replicates for the swelling and gel fraction experiments.

Finally, the capacity of hydrogels to retain moisture was determined according to the protocol adapted from Soler et al. [52]. The initial weight (*W_o_*) of hydrogel samples was recorded before the samples were incubated at 37 °C and relative humidity of 50%. After 2 and 24 h of incubation, the samples were re-weighed (*W_h_*), and the moisture retention capability (*R_h_*) was calculated by the following equation:

*R_h_ (%) = W_h_/W_o_× 100%* (6)

Experiments were performed in 3 replicates at each temperature setting.

### 4.5. Tensile Test

Crosslinked hydrogel sheets were cut into a dumbbell shape according to ASTM D-1822 before being mounted on a texture analyzer (Lloyd LS1, Ametek (GB) Ltd., Leicester, UK). Tensile loading was performed using a 50 N load cell at a crosshead speed of 50 mm/min. At the beginning of each test, the width and thickness of the sample were input into the software (Nexygen V.4.6, Lloyd LS1, Ametek (GB) Ltd., Leicester, UK). Data were collected as load, displacement, stress, and strain. Experiments were performed in 5 replicates.

### 4.6. Coating of ROSE on Hydrogels

A 7.8% (*w*/*v*) solution of ROSE was prepared by dissolving crude extract in DI water. Then, 100 µL of ROSE solution was uniformly coated on PVA hydrogel patches. The ROSE-coated hydrogel patches were finally exposed to electron beam irradiation at 5 and 10 kGy, and decontamination was confirmed by the plate count method.

### 4.7. Morphology Analysis

Non-treated and ROSE-coated hydrogel sheets with thicknesses of about 5 mm were frozen at −80 °C in their swollen state overnight before being lyophilized at −49 °C and 0.08 mbar (Christ Alpha 2-4 LD plus, Germany) for 72 h. The samples were then immersed in liquid N2 for at least 30 min before quickly fracturing. The cut surface was coated with gold–palladium and examined by scanning electron microscopy (SEM, Hitachi SU8020, Japan) at 5 kV and 10.5 µA settings. Assuming a circular shape, each pore was measured from two orthogonal diameters by ImageJ software and then averaged to obtain the pore size. About 250–300 pores were counted for each non-treated and ROSE-coated hydrogel.

### 4.8. In Vitro Release Profile of ROSE from the Hydrogel

The in vitro release of ROSE from hydrogel was carried out in pH 7.4 phosphate buffer and pH 5.5 acetate buffer to represent bodily fluid and skin environment, respectively. The hydrogel was placed in 20 mL of buffer in a flask and kept in a shaking bath (IKA^®^ KS4000i control, Bangkok, Thailand) with a rotation speed of 25 rpm at 37 °C for pH 7.4 phosphate buffer and at 35 °C for pH 5.5 acetate buffer. Samples were withdrawn at predetermined time intervals and replaced with the same volume of fresh buffer. The absorbance of ROSE released from hydrogel was examined using a UV–visible spectrophotometer (UV1800, Shimadzu, Japan) at 360 nm based on our previous study [26]. All experiments were carried out in triplicate (*n* = 3).

*Red onion extract release (% w/w) = (Amount of extract released)/(Amount of extract loaded ) × 100%* (7)

### 4.9. Permeation Study of ROSE in Hydrogel through Newborn Pig Skin 

Permeation study of ROSE-coated hydrogel patches across excised newborn pig skin was determined using a vertical Franz cell and equipment (PermeGear, Inc., Hellertown, PA, USA). One dead newborn pig skin was obtained and frozen within 24 h after death on the farm. After the newborn pig was unfrozen, the skin was separated easily with a razor blade. The newborn pig did not have so much fat under the skin. The remaining fat was removed by the razor blade. The skin was observed visually for skin damage. The receptor cells were filled with 14 mL of pH 7.4 phosphate-buffered saline (PBS) and allowed to equilibrate at 37 °C under magnetic stirring. The skin was cut to fit the vertical diffusion cell’s available area of 1.77 cm^2^. The skin was pre-equilibrated in PBS at room temperature before the experiment for 1 h and was placed by turning the stratum corneum side facing the donor compartment. The hydrogel containing ROSE was placed in the donor compartment. The permeation of ROSE was determined after time intervals of 30 min, and 1, 2, 3, 4, 5, and 24 h by withdrawing 1 mL of the receiving solution and then replacing it with an equal volume of PBS to maintain sink condition. The experiments were performed in triplicate (*n* = 3), and the standard deviation of results was considered. The concentrations of ROSE in the receiver medium were determined by UV–visible spectrophotometry (UV1800, Shimadzu, Japan) at the maximum wavelength of 360 nm. The standard curve was plotted between the absorbance at 360 nm and ROSE concentrations in a range of 10–80 µg/mL. The cumulative amount (µg/cm^2^) of ROSE permeated through the newborn pig skin was calculated and plotted as a function of time.

### 4.10. FTIR Analysis of ROSE-Coated Hydrogel Patch

Red onion skin extract (ROSE) was thoroughly ground to form fine powder prior to analysis. PVA hydrogel and ROSE-coated PVA hydrogel patches were freeze-dried and then characterized as dried sample patches. FTIR measurement (Perkin-Elmer, Waltham, MA, USA) was performed over the range of 4000–550 cm^−1^.

### 4.11. Cell Culture

MEM and DMEM supplemented with 10% FBS, 4 mM L-glutamine, and 100 IU/mL penicillin–streptomycin will be complete MEM and complete DMEM, respectively. The L929 and HDFB were cultured in complete MEM and complete DMEM in a humidified incubator at 5% CO_2_ and 37 °C. The media were replenished daily, and subculture was performed at ~80% confluency. Cells within 12 passages after being purchased were used in all experiments.

### 4.12. In Vitro Cytotoxicity Test by MTT Assay

Cytotoxicity of starting PVA hydrogels and ROSE was determined by the MTT-based extract test. For PVA hydrogels, the samples were first cut into 0.5 cm × 2.5 cm sheets and eluted in complete MEM for 24 h inside a 15 mL centrifuge tube. Negative and positive controls were also cut into 0.5 cm × 2.5 cm sheets and eluted in complete MEM to serve as positive and negative controls, respectively. For ROSE, the extracts were diluted in complete MEM or complete DMEM at concentrations of 0.4, 0.5, 0.6, 0.8, 1.0, and 1.2 mg/mL with 10% (*v*/*v*) DMSO diluted in complete media and blank complete media served as positive and negative controls, respectively.

L929 and HDFB cells were seeded in 96-tissue culture plates at a density of 1 × 10^4^ cells per well in complete MEM and incubated in a humidified incubator at 5 % CO_2_ and 37 °C for 24 h before being refreshed with 100 µL of extracts and further incubated for 24 h. The extracts were then removed, and 50 µL of freshly prepared MTT solution in MEM without phenol red at a concentration of 10% (*w*/*v*) was added to each well. After 3 h of incubation, the resulting formazan crystals formed inside the cells were dissolved in 50 µL of isopropanol. The absorbance of the purple solution was measured with a microplate reader (SpectraMax M3, Molecular Devices, San Jose, CA, USA) at 570 nm. Cell viability was determined based on the absorbance ratio of each sample over the blank control. Experiments were performed in triplicate.

### 4.13. Statistical Analysis

Data were analyzed by GraphPad Prism version 9.2.0 (GraphPad Software, San Diego, CA, USA) and SPSS version 18.0 (SPSS Inc, Chicago, IL, USA). The one-way analysis of variance (ANOVA) and Tukey post hoc test were performed for statistical analysis. To compare the significance of the difference between the means of two groups, a t-test was performed. Results are presented as mean ± standard deviation (S.D.). A *p*-value < 0.05 was considered statistically significant.

## Figures and Tables

**Table 1:** The physical characteristics of 10PVA hydrogels are described by water content, equilibrium degree of swelling (EDS) at 24 h, gel fraction, and moisture retention (*R_h_*). Data are presented as mean ± standard deviation (*n* = 4 for water content, EDS, and gel fraction, and *n* = 3 for *R_h_*).

	Water Content (%)	EDS (%)	Gel Fraction (%)	*R_h_* (%)	
				2 h	24 h
10PVA hydrogel patches	93.0 ± 0.6	1756.0 ± 156.1	89.6 ± 5.8	56.0 ± 8.9	9.6 ± 0.2

**Table 2:** Tensile analysis of 10PVA hydrogel patch cut into dumbbell shape (ASTM D-1822-L) using 50 N load cell at a crosshead speed of 50 mm/min. Data are presented as mean ± standard deviation (*n* = 6).

	Tensile Strength (kPa)	Elongation at Break (%)	Young’s Modulus (kPa)
10PVA hydrogel patches	7.4 ± 1.7	72.3 ± 21.3	11.5 ± 1.4

**Table 3:** Plate count results of non-irradiated and irradiated ROSE-coated hydrogel sheets from 3 different batches.

Samples	Batch 1	Batch 2	Batch 3
Non-irradiated	124	38	682
Irradiated, 5 kGy	0	0	0
Irradiated, 10 kGy	0	0	0
